# Association of vitamin D receptor polymorphisms with the risk of prostate cancer in the Han population of Southern China

**DOI:** 10.1186/1471-2350-10-125

**Published:** 2009-12-04

**Authors:** Yongheng Bai, Yaping Yu, Bin Yu, Jianrong Ge, Jingzhang Ji, Hong Lu, Jia Wei, Zhiliang Weng, Zhihua Tao, Jianxin Lu

**Affiliations:** 1 Zhejiang Provincial Key Laboratory of Medical Genetics, School of Laboratory Medicine and Life Science, Wenzhou Medical College, Wenzhou 325035, PR China; 2 Medical College of Shaoxing University, Shaoxing 312000, PR China; 3 Laboratory Diagnostic Center of the First Affiliated Hospital of Wenzhou Medical College, Wenzhou 325000, PR China; 4 Department of Urology of the First Affiliated Hospital of Wenzhou Medical College, Wenzhou 325000, PR China

## Abstract

**Background:**

Molecular epidemiological studies have shown that gene polymorphisms of vitamin D receptor (*VDR*) are associated with prostate cancer risks. However, previous results from many molecular studies remain inconsistent.

**Methods:**

Blood samples were collected from 122 prostate cancer patients and 130 age-matched control subjects in the Han population of Southern China. The differences of *VDR *gene polymorphism between cancer cases and controls were determined by PCR-RFLP, examiming *Fok*I (exon 2), *Bsm*I, *Tru9*I, *Apa*I (intron 9), and *Taq*I (exon 9). Associations between the *VDR *gene polymorphism and prostate cancer risk were calculated in an unconditional logistic regression model. Linkage disequilibrium and haplotypes were analyzed with the SHEsis software.

**Results:**

Of five polymorphisms, *Bsm*I was shown to associate with prostate cancer, while *Fok*I, *Tru9*I, *Apa*I, and *Taq*I did not show any significant association. After adjustment for age, the *Bsm*I '*B*' allele was associated with an almost 1/3-fold risk (OR = 0.35, 95%CI: 0.15-0.80) of the occurrence of prostate cancer, a 1/5-fold risk (OR = 0.20, 95%CI: 0.06-0.68) of poorly differentiated prostate cancer, and a 1/10-fold risk (OR = 0.10, 95%CI: 0.01-0.78) of aggressive prostate cancer compared with the '*b*' allele, especially among older men (>71 years). In addition, haplotype analysis revealed that the *'F-b-U-A-T*' was more frequent found in cases than in controls (3.4% *vs *0.0%, *P *= 0.0035), while the frequency of haplotype '*F-B-U-a-T*' was 0.8% in cases, significantly lower than in controls (3.9%, *P *= 0.019).

**Conclusion:**

Our experiments provide evidences that genetic polymorphisms in the *VDR *gene may be potential risk factors for prostate cancer in the Han population of southern China and the susceptibility to prostate cancer is associated with ethnicity and geographic location.

## Background

Prostate cancer is the most common type of tumor in old men throughout the world. In 2003, about 220,900 new cases of prostate cancer were diagnosed in the United States [1]. Although epidemiological studies showed that the incidence of prostate cancer in Asians is much lower than that in African-Americans [2], the occurrence of this disease has rapidly increased among Chinese men [3]. In the Shanghai population, the incidence was 1.9/10 million in 1983, but it increased to 21.08/10 million in 2005 [4]. The incidence rate in Beijing was similar to that of Shanghai [5]. Age, race, and geographical factors are well-established risk factors of prostate cancer [6]. However, these factors may not completely explain the differences between different ethnic groups in prostate cancer rates. Therefore, genetic variation in certain genes, including genes controlling vitamin D activity, could play a role in determination of susceptibility to prostate cancer.

Based on investigations of the differences in prostate cancer incidence at different latitudes in the United States, Schwartz et al. [7,8] hypothesized that low ultraviolet exposure may be a risk factor for prostate cancer. After exposure to UV radiation, vitamin D is produced in the skin and then hydroxylated in the liver to 25-hydroxyvitamin D_3_, and in the kidneys to the more physiologically active metabolite 1α,25-dihydroxyvitamin D_3 _[1,25(OH)_2_D_3_]. The biological activity of 1,25(OH)_2_D_3 _is mediated primarily by vitamin D receptor (VDR). In addition to kidney and bone cells, VDR [9] is also expressed in human prostatic cells, and a series of experiments revealed that VDR mediates the influence of 1,25(OH)_2_D_3 _on the growth, differentiation [10,11], and apoptosis [12] of prostatic cells. These findings indicated that 1,25(OH)_2_D_3 _and its specific nuclear receptor protein, VDR, may be closely associated with prostate cancer risk.

**Figure 1:**
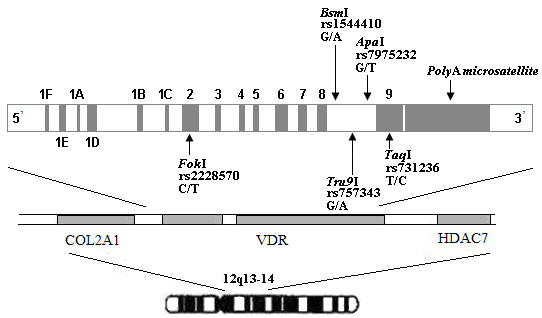
**Positions of polymorphisms in the vitamin D receptor gene**.

The expression and function of VDR may be influenced by *VDR *gene polymorphisms. Polymorphisms (intron 8, exon 9, and polyA microsatellite, Figure 1) in the 3' end may correlate with gene transcription [13]. In exon 2 of 5' end, the *Fok*I *F *allele may lack the first start codon [14], resulting in a VDR protein that is shortened by three amino acids [15]. This protein variant was shown to be more efficient at transactivating a vitamin D-regulated target gene [16]. Polymorphic variants of the *VDR *gene have been suggested to be associated with prostate cancer risk and/or a more aggressive phenotype [17,18]. Gene polymorphisms have been reported to be of significant relevance with cancer risk in many different populations [17-26]. Since there is a series of *VDR *gene polymorphisms that are in strong linkage disequilibrium with one another, some *VDR *haplotypes have also been reported to be linked with prostate cancer [27,28]. However, other studies [29-33] did not show such significant associations. The inconsistent results were probably due to differences in the study populations [20,22].

In this report, we carried out a population-based, case-control study to evaluate the association between *VDR *gene polymorphisms and prostate cancer risk in the Han population of Southern China because one salient characteristic of China's population has a large base with diverse genetic backgrounds. The present study can provide a platform to help explain the pathological mechanism of prostate cancer and to a better understanding of the geographic and ethnic differences associated with disease incidence and mortality.

## Methods

### Subjects

In the present study, 122 prostate cancer patients and 130 age-matched male controls were enrolled in four hospitals in southern China between March 2007 and August 2008. All prostate cancer cases were confirmed by pathological examination. The pathologic grades were classified according to the Gleason score as well and moderately differentiated (Gleason 2-6) and poorly differentiated (Gleason 7-10) [34]. Based on the TNM classification system promulgated by the American Joint Committee on Cancer, the disease stage was divided into localized and aggressive cancer, as determined by transrectal ultrasound, magnetic resonance imaging (MRI) and emission computed tomography (ECT). Localized prostate cancer can be detectable clinically or felt (palpated) on examination, but has not spread outside the prostate. Aggressive cancer means the cancer has spread through the prostatic capsule (eg, bladder neck, external sphincter, rectum, and levator muscles) and even has invaded other nearby structures (eg, bone and pancreas).

The age-matched control subjects were individuals recruited from retired employees at the First Affiliated Hospital of Wenzhou Medical College. The controls were screened to ensure that there had never been diagnosed with cancer and had low plasma prostate-specific antigen (PSA) levels (total PSA<4.00 ng/ml). The controls were also checked for cancer history based on their past medical records and/or asked directly for their cancer history.

All subjects were informed about the contents of the study and gave their informed consent. This study was approved by the Ethics Committee of Wenzhou Medical College.

### Molecular analysis

Blood samples were collected with the anticoagulant EDTA K_2 _and stored at -20°C. Genomic DNA was extracted using a DNA Extraction Kit (TaKaRa Bio Group, Japan) and stored at 4°C. *VDR *polymorphisms were genotyped by PCR-RFLP and the condition was displayed in the Table 1. To control the experiments, a total of 80 samples were randomly selected and genotyped and confirmed by DNA sequencing by a second investigator.

**Table 1:** PCR-RFLP conditions for *VDR *gene polymorphisms (GenBank AY342401)

SNP	Primer	Base change	Annealing temperature	Restriction enzyme
*Fok*I	5'AGCTGGCCCTGGCACTGACTCTGCTCT3'(F)	C/T	61°C	*Fok *I
	5'ATGGAAACACCTTGCTTCTTCTCCCTC 3'(R)			
*Bsm*I	5'CAACCAAGACTACAAGTACCGCGTCAGTGA3'(F)	G/A	57°C	*Mva1269 *I
	5'AACCAGCGGGAAGAGGTCAAGGG3'(R)			
*Tru9*I	5'CAACCAAGACTACAAGTACCGCGTCAGTGA3'(F)	G/A	57°C	*Tru1 *I
	5'AACCAGCGGGAAGAGGTC AAGGG3'(R)			
*Apa*I	5'CAGAGCATGGACAGGGAGCAA3'(F)	G/T	60°C	*Apa *I
	5'GCAACTCCTCATGGCTGAGGTCTC3'(R)			
*Taq*I	5'CAGAGCATGGACAGGGAGCAA3'(F)	T/C	60°C	*Taq *I
	5'GCAACTCCTCATGGCTGAGGTCTC3'(R)			

*Fok*I, *Bsm*I, *Tru9*I, *Apa*I, and *Taq*I, are reported according to the standard nomenclature in which lowercase and uppercase letters indicate the presence or absence of a restriction site, respectively. The *Fok*I *T *and *C *alleles are represented by *f *and *F*, the *Bsm*I *G *and *A *alleles by *b *and *B*, the *Tru9*I *G *and *A *alleles by *U *and *u*, the *Apa*I *T *and *G *alleles by *A *and *a*, and the *Taq*I *T *and *C *alleles by *T *and *t*, respectively.

### Statistical analysis

Tests for Hardy-Weinberg equilibrium were performed separately for each SNP among case and control subjects. The Independent-Samples T test was used to determine differences according to age, and the chi-square or Fisher's exact test was performed to calculate the clinical parametric distributions. Unconditional logistic regression analysis models were used to evaluate the relationships between different genotypes and disease risk [Odds ratios (OR), 95% confidence intervals (95% CI)] adjusted by age. Linkage disequilibrium and haplotypes were analyzed with the SHEsis software [35]. A *p*-value of less than 0.05 was considered as statistically significant.

## Results and Discussion

The worldwide incidence of clinical prostate cancer shows a strong dependence on age, race, and geography. For example the incidence in African-Americans was significantly higher than that in Asians, especially in the Chinese population. Recent epidemiological studies in different populations have indicated that difference in *VDR *genotype frequencies may play an important role in the risk of prostate cancer. In the present study, we evaluated the association between five polymorphisms in the *VDR *gene and prostate cancer risk in the southern Chinese Han population.

Table 2 shows the demographic and clinical characteristics of the subjects (122 prostate cancer patients and 130 non-cancer controls). No significant differences in age, smoking status, or alcohol intake were observed between cases and controls. In the present study, five polymorphisms, including *Fok*I at the 5' end of exon 2, and *Bsm*I, *Tru9*I, *Apa*I, and *Taq*I in the 3'UTR region, were evaluated, and each SNP was in Hardy-Weinberg equilibrium. Statistical analysis revealed a significant difference in the allelic frequencies of the *Bsm*I genotype (Table 3), while other polymorphisms did not show any significant differences. Compared with *Bsm*I '*bb*', subjects with *'BB*' and *'Bb*' genotypes were associated with a decreased risk (OR = 0.35, 95%CI: 0.15-0.81, *P *= 0.015). The *Bsm*I *'B*' allelic frequency was 3.7% in cancer patients, which was significantly lower than that of controls (8.5%), indicating a decreased disease risk associated with this allele (OR = 0.35, 95%CI: 0.15-0.80, *P *= 0.013). To evaluate the association modified by the age, we stratified the age to two groups (>71 and ≦71 years). Among the elderly men, the OR value for the "*B*" allele was 0.09 (95%CI: 0.01-0.68) versus the "*b*" allele (Table 4), and the OR value was 0.64 (95%CI: 0.25-1.66) among the younger men, suggesting that the elder men with the "*B*" allele had lower risk of prostate cancer. In addition, when stratifying our material according to Gleason score and TNM classification, a number of significant results were found. The '*B*' allele (compared to the '*b*' allele) was also associated with a decreased risk of aggressive (OR = 0.10, 95%CI: 0.01-0.78, *P *= 0.028) and poorly differentiated cancer (OR = 0.20, 95%CI: 0.06-0.68, *P *= 0.010, Table 4). Considering the number of cases with the variant allele for most of these strata is too small, and future research needs to further expand the sample size to confirm these results.

**Table 2:** Clinic and demographic characteristics of the subjects

	Cases (n = 122)	Controls (n = 130)	OR(95%CI)	*P *value
Ages at diagnosis (year)^a^	71.77 ± 8.12	71.28 ± 7.86		0.632
< 60	11 (9.0%)	11 (8.5%)		
60~69	33 (27.1%)	42 (32.3%)		
70~79	56 (45.9%)	59 (45.4%)		
≥ 80	22 (18.0%)	18 (13.8%)		
Smoking status ^b^				
Nonsmoking	82 (67.2%)	91(70.0%)	1.138(0.668-1.939)	0.634
Smoking	40 (32.8%)	39(30.0%)		
Alcohol intake ^b^				
No alcohol	62 (50.8%)	69 (53.1%)	1.095(0.668-1.795)	0.720
Drinking	60 (49.2%)	61 (46.9%)		
Gleason score				
< 7	43 (35.2%)			
≥ 7	79 (64.8%)			
TNM classification				
Localized	72 (59.0%)			
Aggressive	50 (41.0%)			

^a^, Data are expressed as mean ± standard deviation (SD), *P *values are calculated using unpaired t-test;

^b^, Based on chi-square test.

**Table 3:** Distribution of *VDR *genotypes and alleles between prostate cancer cases and controls

		Cases (freq)	Controls (freq)	OR(95%CI)	*P *value
*Fok*I	*FF*	33 (0.270)	38 (0.292)	1.00(referent)	
	*Ff*	63 (0.517)	55 (0.423)	1.317(0.730-2.376)	0.361
	*ff*	26 (0.213)	37 (0.285)	0.815(0.411-1.620)	0.560
	*F*	129 (0.529)	131 (0.504)	1.00(referent)	
	*f*	115 (0.471)	129 (0.496)	0.910(0.641-1.291)	0.597
*Bsm*I	*bb*	114 (0.934)	108 (0.831)	1.00(referent)	
	*Bb*	8 (0.066)	21 (0.162)	0.362(0.154-0.853)	0.020
	BB *	0 (0)	1 (0.008)	-	-
	*b*	236 (0.964)	237 (0.912)	1.00(referent)	
	*B*	8 (0.036)	23 (0.088)	0.351(0.154-0.800)	0.013
*Tru9*I	*UU*	76 (0.623)	78 (0.600)	1.00(referent)	
	*Uu*	41 (0.336)	47 (0.362)	0.902(0.533-1.525)	0.699
	*uu*	5 (0.041)	5 (0.038)	1.039(0.289-3.742)	0.953
	*U*	193 (0.791)	203 (0.781)	1.00(referent)	
	*u*	51 (0.209)	57 (0.219)	0.947(0.618-1.452)	0.804
*Apa*I	*AA*	10 (0.082)	9 (0.069)	1.00(referent)	
	*Aa*	56 (0.459)	61 (0.469)	0.800(0.300-2.128)	0.654
	*aa*	56 (0.459)	60 (0.462)	0.810(0.304-2.160)	0.673
	*A*	76 (0.311)	79 (0.304)	1.00(referent)	
	*a*	168 (0.689)	181 (0.696)	0.956(0.654-1.397)	0.814
*Taq*I	*TT*	112 (0.918)	121 (0.931)	1.00(referent)	
	*Tt*	10 (0.082)	9 (0.069)	1.213(0.475-3.098)	0.687
	*tt*	0 (0)	0 (0)	-	-
	*T*	234 (0.959)	251 (0.965)	1.00(referent)	
	*t*	10 (0.041)	9 (0.035)	1.204(0.480-3.016)	0.693

*, Genotype (*BB*+*Bb*) *vs bb*, OR _age-adjusted _= 0.346, 95%CI: 0.148-0.811, *P *= 0.015.

**Table 4:** Age-adjusted ORs and *P *value in different kinds of patients compared with controls by *Bsm*I polymorphisms

		Pathological grade				Clinic stage				Age stratification (years)*			
				
	Controls	Well-mod	OR(95%CI)	Poorly	OR(95%CI)	Localized	OR(95%CI)	Aggressive	OR(95%CI)	>71	OR(95%CI)	≦71	OR(95%CI)
*bb*	108	38	1.00(referent)	76	1.00(referent)	65	1.00(referent)	49	1.00(referent)	62	1.00(referent)	52	1.00(referent)
*Bb*	21	5	1.44(0.51-4.11)	3	0.20(0.06-0.71)	7	0.56(0.23-1.40)	1	0.11(0.01-0.81)	1	0.08(0.01-0.64)	7	0.73(0.26-2.04)
*BB*	1	0		0		0		0		0		0	
*Bb+ BB*	22	5	0.97(0.93-1.02)	3	0.19(0.06-0.67)	7	0.54(0.22-1.33)	1	0.10(0.01-0.77)	1	0.08(0.01-0.64)	7	0.67(0.25-1.85)
b	237	81	1.00(referent)	155	1.00(referent)	137	1.00(referent)	99	1.00(referent)	125	1.00(referent)	109	1.00(referent)
*B*	23	5	0.97(0.94-1.00)	3	0.20(0.06-0.68)	7	0.53(0.22-1.28)	1	0.10(0.01-0.78)	1	0.09(0.01-0.68)	7	0.64(0.25-1.66)

*, Stratified by the average age of 71 years.

Based on observations of the distribution difference between prostate cancer cases and controls, we here hypothesize the *Bsm*I '*B*' allele might have a protective effect against tumorigenesis. Previous studies also found that the *Bsm*I *'BB+Bb*' genotype was associated with one-third the risk of prostate cancer in a Japanese population [20] and one-fifth the risk in advanced stages and in poorly differentiated disease in a Taiwanese population [36]. Although Ma et al. [37] reported no significant association between the *Bsm*I polymorphism and prostate cancer risk, they did find a 57% reduction in the risk of prostate cancer with the *Bsm*I '*BB*' genotype compared with the '*bb*' genotype in American men. These data also support our findings concerning the protective effect of the *Bsm*I '*B*' allele. However, there are still some studies failed to show such a result. For example, studies such as Liu et al. [32] in Beijing population and Ingles et al. [28] in African-Americans. In addition to ethnic characteristics, geographical differences and living habits, the factors, including the study sample size, the choice of the control group (BPH or healthy people), might be the important reasons for these inconsistent results.

Although the *Bsm*I polymorphic variant is in the untranslated intron 8 and does not seem to alter the VDR amino acid sequence, some studies have reported that the *Bsm*I '*BB*' genotype is significantly associated with higher plasma 1,25(OH)_2_D_3 _(median = 36.2 pg/ml for the '*BB*' versus 33.9 pg/ml for the '*bb' *genotype; *P *= 0.02), suggesting that the *Bsm*I polymorphic variant is associated with VDR function [37]. In fact, strong linkage disequilibrium was detected among four polymorphisms, including *Bsm*I in the 3'UTR region (Table 5), which is consistent with other studies [13,18,36,38]. In addition, the effect of *Bsm*I may be influenced by other polymorphisms.

**Table 5:** Linkage disequilibrium test between five *VDR *gene polymorphisms

SNP1	SNP2	Distance between two polymorphisms	D'	*P *value^a^
*Fok*I	*Bsm*I	33060	0.513	0.110
*Fok*I	*Tru9*I	33220	0.096	0.803
*Fok*I	*Apa*I	34058	0.169	0.379
*Fok*I	*Taq*I	34138	0.299	0.739
*Bsm*I	*Tru9*I	160	0.574	0.253
*Bsm*I	*Apa*I	998	0.556	0.006
*Bsm*I	*Taq*I	1078	0.826	<0.001
*Tru9*I	*Apa*I	838	0.831	<0.001
*Tru9*I	*Taq*I	918	0.953	<0.001
*Apa*I	*Taq*I	80	0.900	<0.001

^a^, Based on chi-square test.

Haplotypes with five-locus (*Fok*I, *Bsm*I, *Tru9*I, *Apa*I, and *Taq*I) of *VDR *gene polymorphisms were also analyzed with SHEsis software. We found that the haplotype *'F-b-U-A-T*' was more frequent in cases than in controls (3.4% *vs*. 0.0%, *P *= 0.0035; Table 6), while the frequency of haplotype '*F-B-U-a-T*' was 0.8% in cases, significantly lower than that in controls (3.9%, *P *= 0.019), indicating that the haplotype *'F-b-U-A-T*' was associated with increased risk of prostate cancer and the '*F-B-U-a-T*' with a decreased risk. In Caucasian and African-Americans, an association was observed between disease risk and the four-locus '*F-B-A-t*' haplotype (OR = 0.48, 95%CI: 0.30-0.76) compared with the '*F-b-a-T*' haplotype, as reported by Cicek et al. [27]. The haplotype results obtained in that study also partly support our conclusion. In addition to *Bsm*I, linkage disequilibrium data also showed that other polymorphisms (*Tru9*I, *Apa*I, and *Taq*I) may play some role in disease risk through their interactions. Taylor et al. [17] reported that the *Taq*I '*tt*' genotype is significantly decreased in prostate cancer patients (8%) compared to noncancer controls in Caucasian Americans (22%; OR = 0.32; 95%CI: 0.15-0.75). A study of a southern European population also showed an association of the *Taq*I '*T *'allele with cancer risk [22]. However, our study showed that these polymorphisms demonstrated no significant differences in the southern Chinese Han population, which also suggested that the distribution of *VDR *gene polymorphisms may be associated with ethnicity and/or location.

**Table 6:** Haplotype frequencies for *VDR *five polymorphisms between prostate cancer cases and controls

Haplotypes	Controls (freq)	Cases (freq)	*P *value^a^	OR (95%CI)
*F-b-U-A-T*	0.00(0.000)	8.90(0.034)	0.0035	-
*F-b-U-a-t*	6.98(0.029)	5.29(0.020)	-	-
*F-b-U-a-T*	0.00(0.000)	1.94(0.007)	-	-
*F-B-u-A-T*	6.31(0.026)	4.31(0.017)	-	-
*F-B-u-a-T*	18.06(0.074)	17.75(0.068)	0.806	0.92 (0.46~1.82)
*F-B-U-A-T*	87.01(0.357)	90.69(0.349)	0.863	0.97 (0.67~1.41)
*F-B-U-a-T*	9.56(0.039)	2.03(0.008)	0.019	0.19 (0.04~0.89)
*f-B-u-a-T*	0.00(0.000)	1.65(0.006)	-	-
*f-B-U-a-t*	0.00(0.000)	2.66 (0.010)	-	-
*f-b-U-a-T*	0.00(0.000)	2.48(0.010)	-	-
*f-B-u-A-T*	1.54(0.006)	0.00(0.000)	-	-
*f-B-u-a-T*	25.09(0.103)	33.21(0.128)	0.376	1.28 (0.74~2.23)
*f-B-U-A-T*	72.11(0.296)	77.10(0.297)	0.970	1.01 (0.68~1.49)
*f-B-U-a-t*	1.00(0.004)	1.05(0.004)	-	-
*f-B-U-a-T*	14.32(0.059)	10.86 (0.042)	0.385	0.70 (0.31~1.57)
*F-B-U-a-t*	1.00(0.004)	0.00(0.000)	-	-
*f-b-U-A-t*	0.92(0.004)	0.00(0.000)	-	-
*f-B-u-A-T*	1.54(0.006)	0.00(0.000)	-	-

^a^, Based on Fisher's exact test;

All those frequency <0.10 will be ignored in analysis.

The distribution of polymorphisms in the 3'UTR region of the *VDR *gene may be relavant with *VDR *mRNA stability and gene transcription [13]. Alterations in the ability of VDR protein to bind 1,25(OH)_2_D_3 _or activate the *VDRE *gene would ultimately result in changes in the expression of regulatory genes, such as *CDK*, which can directly or indirectly control prostatic cell division. If the protective action of the *Bsm*I '*B*' allele is confirmed experimentally, the pathological mechanism of prostate cancer would be better understand. In addition, we observed the genotype and allelic frequencies of *VDR *gene varied from race to race. For example, the allelic frequency of *Taq*I '*T*' in the southern Chinese Han population (96.5%, in control) was significantly higher than that in white Americans (55.2%) [17] and Portuguese (57.8%) [22]. Thus, the distribution of *VDR *gene polymorphisms may help explain the difference in incidence of prostate cancer in different populations.

## Conclusion

In conclusion, the present study indicated that *VDR *gene polymorphisms may be associated with prostate cancer risk in the southern Chinese Han population, and these associations may largely depend on population characteristics and geographic location.

## Abbreviations

All abbreviations are defined in the text.

## Competing interests

The authors declare that they have no competing interests.

## Authors' contributions

YHB, YPY and JXL conceived the study, designed and coordinated it, performed part of the molecular genetic studies, data analysis, and wrote the manuscript. WJ participated in the molecular genetic studies. BY, JRG, JZJ, HL, ZLW and ZHT participated in the recruitment of prostate cancer cases and control subjects. All authors read and approved the final manuscript.

## Pre-publication history

The pre-publication history for this paper can be accessed here:

http://www.biomedcentral.com/1471-2350/10/125/prepub
